# A Comparison of Machine Learning Classifiers for Energy-Efficient Implementation of Seizure Detection

**DOI:** 10.3389/fnsys.2018.00043

**Published:** 2018-09-20

**Authors:** Farrokh Manzouri, Simon Heller, Matthias Dümpelmann, Peter Woias, Andreas Schulze-Bonhage

**Affiliations:** ^1^Epilepsy Center, Faculty of Medicine, University of Freiburg Medical Center, Freiburg, Germany; ^2^BrainLinks-BrainTools Cluster of Excellence, University of Freiburg, Freiburg, Germany; ^3^Department of Microsystems Engineering, Faculty of Engineering, University of Freiburg, Freiburg, Germany

**Keywords:** intracranial EEG, closed-loop intervention, seizure detection, epilepsy, machine learning, low power microcontroller implementation

## Abstract

The closed-loop application of electrical stimulation via chronically implanted electrodes is a novel approach to stop seizures in patients with focal-onset epilepsy. To this end, an energy efficient seizure detector that can be implemented in an implantable device is of crucial importance. In this study, we first evaluated the performance of two machine learning algorithms (Random Forest classifier and support vector machine (SVM)) by using selected time and frequency domain features with a limited need of computational resources. Performance of the algorithms was further compared to a detection strategy implemented in an existing closed loop neurostimulation device for the treatment of epilepsy. The results show a superior performance of the Random Forest classifier compared to the SVM classifier and the reference approach. Next, we implemented the feature extraction and classification process of the Random Forest classifier on a microcontroller to evaluate the energy efficiency of this seizure detector. In conclusion, the feature set in combination with Random Forest classifier is an energy efficient hardware implementation that shows an improvement of detection sensitivity and specificity compared to the presently available closed-loop intervention in epilepsy while preserving a low detection delay.

## Introduction

### Problem Definition

Epilepsy is one of the most common neurological diseases and it affects almost 70 million people worldwide (Ngugi et al., 2010). Epileptic seizures are defined as episodes of excessive or abnormal synchronous neuronal activity in the brain. Seizures can be accompanied by clinical neurological symptoms, such as abnormal movements, abnormal sensory phenomena, loss of consciousness, or alterations in consciousness, and are therefore associated with considerable neurological morbidity. Seizures can vary widely among patients, and even within individual patients.

Despite progress in the development of medication, about 30 percent of patients are resistant to treatment with antiepileptic drugs (Kwan and Brodie, 2000). Only 7%–8% of these patients can be cured by surgery (Litt and Echauz, 2002). In the case of focal seizures, surgical resection of the region(s) of the brain generating the seizures can be used to prevent further seizures. However, since not all of these patients have a unifocal seizure onset zone (SOZ), and the epileptogenic brain area cannot always be resected without a significant functional deficit, the need for innovative new therapeutic approaches is evident.

A novel approach for patients with drug resistant epilepsy is the application of electrical stimulation to terminate the seizures in their early stages, which is done by means of an implanted device. This approach requires the early detection of seizures with high accuracy based on the intracranial recording of brain generated electric field potentials, called electroencephalography (EEG). For long term applications in an implantable device, the selected seizure detector should have low energy consumption. An overview on practical developments concerning neurostimulation for the treatment of epilepsy is illustrated in Fisher and Velasco (2014) and Schulze-Bonhage (2017). Currently, there is one FDA-approved responsive neurostimulation implant device that is available for clinical applications (RNS System; Sun and Morrell, 2014). Responsive neurostimulation here means responding to a detected seizure with stimulation of the brain. Although this device has proven efficacy both under short-term and long-term application (Heck et al., 2014), it suffers from a high number of false detections, putting into question how much of this effect is due to closed-loop suppression of seizure-related ictal activity. Improved approaches with higher specificity of interventions are thus required.

### Previous Studies

The development of seizure detection algorithms based on EEG started decades ago (Gotman, 1982) with the objective of reducing the workload of reviewing continuous long term recordings in epilepsy monitoring units and presenting only intervals with highest clinical relevance to the neurologist. Due to the high variation of the EEG patterns characterizing a seizure (Meier et al., 2008) and the huge variability of background EEG activity among patients, and intra-individual fluctuations in EEG activity the problem of seizure detection remains an active topic of research (Ramgopal et al., 2014).

New approaches aim to trigger interventions to prevent the occurrence (Mormann et al., 2007) or the spread of seizure activity during the early stage of the seizure. These application scenarios require online applicability at reasonable computational costs. The selection of optimal features for online seizure detection in scalp EEG has been addressed in a study by Logesparan et al. (2012). In order to be able to perform an intervention exactly at the onset of a seizure, early detection is of major importance, which has been taken into account in recent approaches (Donos et al., 2015; Baldassano et al., 2017). Low computational costs, which result energy efficiency, as a prerequisite for the application of seizure detection in implantable devices have to be considered. Early translations to hardware realizations can be found in Salam et al. (2011) and Do Valle et al. (2016).

### Our Approach

The goal of our study is to provide a low power seizure detector that is suitable for online intervention in epileptic brain activity at the early stages of a seizure. To this end, we implemented two detectors based on powerful machine learning algorithms. One detector is based on support vector machines (SVMs) and the other is based on our previously suggested Random Forest classifier (Donos et al., 2015, 2018). We compared the performance of these two classifiers with the seizure detection method implemented in the only FDA-approved implant device for epilepsy patients (Sun and Morrell, 2014) based on thresholding the line length of the recorded signal. We used line length because not only because it is one of the features which has frequently been suggested for seizure detection (Esteller et al., 2001; Logesparan et al., 2012), but also, because it is sensitive to both, frequency and amplitude changes. For both Random Forest and SVM classifiers, an identical feature set was used in order to allow for a comparison of their performance. Then we implemented the classifier with superior performance on the micro-controller to evaluate its efficiency for a later implementation in a closed-loop system. To the best of our knowledge, this is the first study which considers detection delay and energy efficiency at the same time with other seizure detector parameters, and does the comparison with a method used by the only available medically approved responsive neurostimulator.

## Materials and Methods

### Dataset

This study was based on the long-term intracranial: (i) EEG recordings of 10 patients from the European Epilepsy Database (Ihle et al., 2012). Patients had undergone presurgical evaluation using implanted strip, grid, or depth electrodes, allowing to record subdurally from the cortical surface and from structures below like cortical sulci, hippocampus and amygdala. iEEG was usually recorded over a period of 1 to 2 weeks. Recorded ictal and interictal iEEG data during this time period were used for classifier training. For a robust validation of the seizure detection algorithm, we selected patients *a priori* with different seizure onset patterns (EEG patterns at the early stages of a seizure e.g., rhythmic spiking or rhythmic beta activity). The 10 analyzed patients had a total number of 160 clinical seizures, annotated by experienced epileptologists. The patients had between 6 and 26 seizures. The SOZ was identified by the gold-standard, which is visual analysis of intracranial EEG by experienced epileptologists. We selected the minimum number of channels in the SOZ so that each recorded seizure had its onset in at least in one of the selected channels; only these channels were used for the classifier training and testing. The study was designed in two modes: single channel and multichannel. In the single channel mode, classification was performed separately on each of the selected channels from the SOZ (single channel classifier). In the multichannel mode, four channels from the SOZ were used for training and testing. The EEG recordings were split into 1-h segments, each containing at least one seizure. In some blocks there were two or three seizures, so these blocks were divided such that in every block there was exactly one seizure. Epochs were labeled as seizure or not seizure based on the electrographical seizure onset and end. Since the ratio between seizure and non-seizure data is very unbalanced in 1-h blocks, the remaining non-seizure hours, which would even worsen this ratio, were not used. The recordings were locally re-referenced by subtracting the average of all channels of one electrode (depth or grid) from each channel of the same electrode.

The EEG-data were obtained using a Neurofile NT digital video-EEG system with 128 channels at a sampling rate between 256 Hz and 1,024 Hz, and a 16-bit analog-to-digital converter (ADC). The signal was filtered in the recording system with a high-pass filter with a time constant of 1 s and a low-pass filter with a cutoff frequency of 344 Hz for recordings with 1,024 Hz (respective 90 Hz for recordings with 256 and 512 Hz sampling frequency). For faster and more energy efficient computation, the recordings were re-sampled to 256 Hz for recordings with original sampling frequencies of 512 Hz and 1,024 Hz.

Since the algorithm is aimed to be used for seizure intervention, the ability to detect the seizure onset at the early stages is of crucial importance, as it increases the chance of an intervention prior to a clinical manifestation. Among the different parameters, the seizure detection time window, which is given by the duration of the EEG data epochs used for feature calculation, affects the seizure detection delay. To obtain the chance of early detection without the use of overlapping windows, which introduces extra computational demands, we performed an analyses based on non-overlapping time windows of 1 s.

### Data Preprocessing

To improve the quality of the data and suppress artifacts before feature extraction, preprocessing steps were performed. First, to remove the slow drifts, data were filtered with a high-pass filter of 0.5 Hz. Then, a band-stop filter was used to remove the powerline noise. Subsequently, to remove data epochs with degraded quality due to artifacts, an artifact rejection step was added. This artifact rejection algorithm rejected epochs with very low variance (compared to the mean variance) and epochs with either very high or extremely low amplitude. Finally, since some patients with short intracranial bursts, that have high-frequency (>50 Hz) content, cause a high number of false positive detections, a low-pass sixth order Butterworth filter (with 50 Hz cut-off frequency) was used to remove activity in these frequency bands.

### Line Length Based Classifier

The first detector investigated in this study is based on line length. This is a signal feature calculated with low computational power, and is defined as the total length of the curve or sum of distances between successive points (Equation 1; Esteller et al., 2001).

(1) $$L=\sum_{i=1}^{N}abs[x\left(k-1\right)-x(k)]$$

In this study, we normalized the line length by z-scoring. Mean and standard deviation were calculated for each seizure separately based on the related 1-h data segment. In the multichannel mode, a logical “or” function was used to combine the results of the four detection channels.

### Features Set for Random Forest and SVM Classifiers

Ten time and frequency domain features were selected based on a reasonable computational demand. Time domain features were mean, mean absolute deviation, variance, skewness, kurtosis, line length and autocorrelation. Frequency domain features included average power (MATLAB Signal Processing Toolbox, 2017) in beta (13–30 Hz) and gamma (30–50 Hz) bands, and a power-ratio between the alpha (7–13 Hz), beta and gamma bands. This power-ratio is defined as the power in the gamma band divided by the sum of the power in the alpha and beta bands; its selection is based on an increase in gamma band power and a decrease in alpha and beta band power in a frequent seizure onset pattern, low voltage fast activity (LVFA), resulting in higher power-ratio values at the seizure onset. The feature definition is also close to the first stage of the computation of the epileptogenicity index (Bartolomei et al., 2008), which consists of the signal energy between high (beta and gamma) and low (alpha and theta) frequency bands of the EEG. The ratio used in this study is potentially even more sensitive for high frequency activity than the power ratio used for the computation of the epileptogenicity index.

### Random Forest Classifier

The second seizure detection algorithm uses the Random Forest (Breiman, 1996, 2001) approach for classification. The Random Forest is an ensemble learning method for classification or regression that operates by constructing a group of decision trees where each tree is grown using binary decisions (each parent node is split into two children). The classifier combines the “bagging” technique with random selection of features. The randomness of each tree is realized in two ways: first by selecting a subset of about two thirds of the data for training, and second by feature selection for nodes of each tree, which is done from a subgroup of features selected randomly. The remaining one third of the training data is used for out-of-bag error evaluation and to calibrate the performance of each tree. The branching index for growing each decision tree of the Random Forest is the so-called Gini index. To define, Gini index is a measure of how often a randomly chosen element from the set would be incorrectly labeled, if it was randomly labeled in accordance with the distribution of labels in the subset. The best feature (splitter) from the eligible random features subset that has the most importance is used to split the node. The importance of each feature is computed based on the decreasing of the Gini index. For this study, the number of binary decision trees in a Random Forest was set to 100 (Donos et al., 2015). Higher numbers of trees were tested, but they did not improve the accuracy of the classification significantly. According to the Liaw and Wiener (2002) results, the optimal number of features randomly selected at each tree node is √N, where N is the number of features. In our case we have ten features and we rounded up the squared number of features, resulting in four features randomly selected at each node.

For classification we used Liaw and Wiener (2002) implementation of Random Forest. To evaluate the performance of the Random Forest classifier we used the “leave-one-out” method as a cross validation method since numbers of seizures for some patients were low. It means that for each of the data points in the set, the function approximator is trained on all the data, except for that one data point and a prediction is made for that point. The average error is computed and used to evaluate the model. The implemented MATLAB code for the feature calculation and random forest seizure detector for interested readers are available per request by sending an email to the first author.

### Support Vector Machines (SVM)

The third algorithm which we implemented for seizure detection is based on a SVM classifier. The basic idea of the SVM is to find the optimal hyperplane for linearly separable patterns (Müller et al., 2001). The optimization is done by finding the hyperplane that represents the largest separation (margin) between the two classes. SVM also extends to patterns that are not linearly separable by transformations of original data into a new space using kernel function. Kernel function maps data onto a richer feature space where the patterns are separable, using a linear hyperplane.

We used the same set of features for classification as for the Random Forest classifier to allow for a comparison of performance measures. Due to the fact that the range of features affects their weight and consequently decision boundaries in SVM, feature values were normalized before the classification stage. This contrasts to the decision trees of the Random Forest classifier where one feature is never compared in magnitude to other feature ranges, and thus the range of the features does not affect the decision boundaries. Without normalizing, large attribute values might furthermore cause numerical problems as kernel values usually depend on the inner products of feature vectors (Hsu et al., 2016).

We used the Radial Basis Function (RBF) as the kernel function to handle the nonlinearity between the features and class labels. An advantage of RBF is the low complexity of the model selection because of the low number of hyperparameters to be optimized in addition few numerical difficulties (Chang and Lin, 2011).

For the SVM classifier, there are two optimization parameters. One is γ of a Gaussian function (Equation 2). The other is C, which is the penalty parameter of the error term and determines how relaxed the margins are (Hsu et al., 2016). We did a grid search for every detection electrode to ensure the best classification parameters for every data channel. To predict the accuracy of the classifier in the parameter optimization step and to prevent the over fitting problem, a 5-fold cross-validation was done on the training dataset. We decided to do a 5-fold cross-validation because the runtime required for optimization was very long and some patients had a high number of seizures. The cross-validation was performed to find the best (highest accuracy) parameter C and γ for training the classifier.

(2) $$K\left(x,y\right)=\;e^{-\gamma {\left|x-y\right|}^{2}}$$

For training and testing, we used the LIBSVM python toolbox for our implementation (Chang and Lin, 2011). LIBSVM uses a sequential minimal optimization (SMO) algorithm to solve the quadratic minimization problem as convergence method. For a faster convergence of the classifier the shrinking technique was applied. The shrinking technique reduces the size of the problem by temporarily reducing the number of variables that have to be updated in each iteration in the process of choosing the search direction (Joachims et al., 1999; Bottou and Lin, 2007). To evaluate the performance of SVM classifier we used “leave-one-out” method as cross validation method since number of seizures for some patients is low.

### Hardware Implementation of the Seizure Detector on a Microcontroller

The optimization goal of a neuro-implant is a low power consumption to increase battery lifetime and to guarantee that the surrounding tissue does not warm up due to dissipated power. Therefore, a 16-bit low power microcontroller from Texas Instruments (MSP430FR5994) was chosen to implement the feature extraction and the classification.

The biggest challenge of the hardware implementation is the limited resolution from the 16-bit architecture yielding truncations errors. For efficient implementation of feature extraction primarily using the hardware multiplier in combination with the direct memory access (DMA) controller and the integrated low power accelerator for digital signal processing (DSP), a 10-bit resolution for the ADC is chosen. To compare the performance of the hardware implementation, first it is simulated with MATLAB. The electrode signals were scaled by a gain factor calculated from Equation 3 to simulate the later analog part of the implant consisting of a low power amplifier and the microcontroller’s internal ADC. Instead of scaling to the min/max values of the electrode signals, +/− 10 times the standard deviation of the signal was chosen to suppress artifacts and amplify the useful signal as high as possible.

(3) gain=(20×std(testData)×resolution ADC)/reference voltage ADC

The algorithm of the seizure detector consists of feature extraction and classification. Both parts have to be executed on the microcontroller in parallel to the data acquisition of the electrode signals. Using combination of a timer triggering the ADC and a DMA controller transferring the ADC’s output to memory, a data acquisition without intervention of the CPU was implemented. Analyzing the required mathematical operations for feature extraction made it apparent that for the time domain feature, an efficient multiply and add (MAC) operation is required and for the frequency domain feature, an efficient FFT algorithm is required. An efficient MAC operation can be achieved using the hardware multiplier to trigger two DMA channels to load new input data. Again, the CPU is not required and can be turned off to save one third of the power consumption. This is also the case for the calculation of the FFT, as the microcontroller has a DSP accelerator for this task.

### Performance Analysis

In order to compare the performance of the optimization techniques, we considered the following three parameters: seizure detection delay, sensitivity and False Detection Rate (FDR). Seizure detection delay is the time between the first ictal activity, that has been correctly detected and the labeled onset of electrographic ictal activity in seconds. Since data is divided into 1-s epochs, if the first second of ictal activity is correctly detected, delay is 1 s. Therefore, the minimum achievable onset detection delay is 1 s. Sensitivity (true positive rate) is a measure of the ability of the classifier to detect seizures and avoid false negative detections. It is defined as the ratio of correctly detected seizures to the total number of seizures. A seizure is detected correctly when at least one electrode “detects” a seizure at least once during the ictal phase. FDR is a tool for measuring the ability of the classifier to avoid false positive detections, which is defined as the number of false detections made by the classifier in an hour. Seizure-onsets are defined by board-certified epileptologists based on emergence of ictal patterns in the EEG data. Due to the fact that seizure patterns show a gradual evolution of hypersynchronous activity with recruitment of an increasing number of neurons, judgments on the definite appearance of a seizure pattern can vary by several seconds. Regarding this existing inter-rater variability between epileptologists in the seizure onset labeling, we considered a detection made in the 0–5 s interval prior to the seizure onset as correct detection with zero delay. Besides, possible subtle changes in the EEG that were not visible without numerical analysis may introduce some degree of uncertainty about the exact seizure onset point.

## Results

### Comparison of Classifier Performance

**Figure 1:**
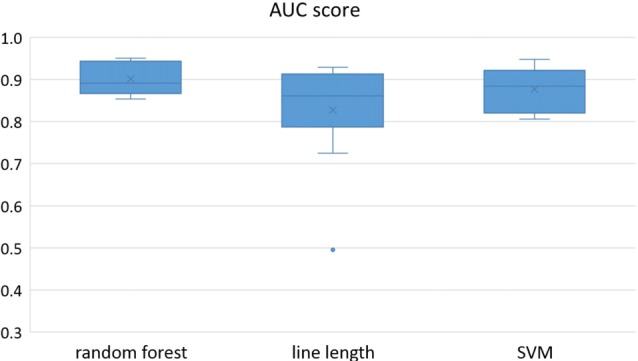
Detection performance of the classifiers over all the patients based on a best channel (highest area under the receiver operating characteristic (ROC) curve (AUC)) selection per patient.

**Figure 2:**
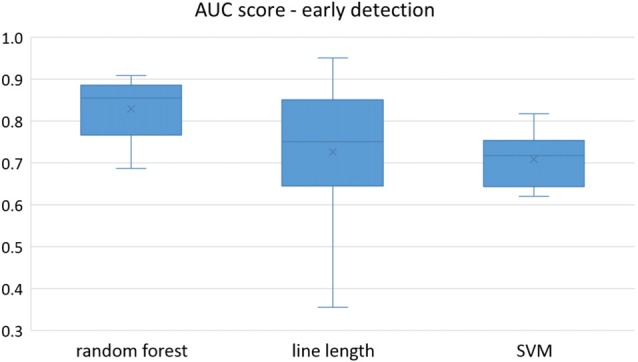
Early detection performance of the classifiers over all the patients based on a best channel (highest AUC) selection per patient. Here, just the seizures which could be detected in the first 10 s are counted as a true positive.

**Figure 3:**
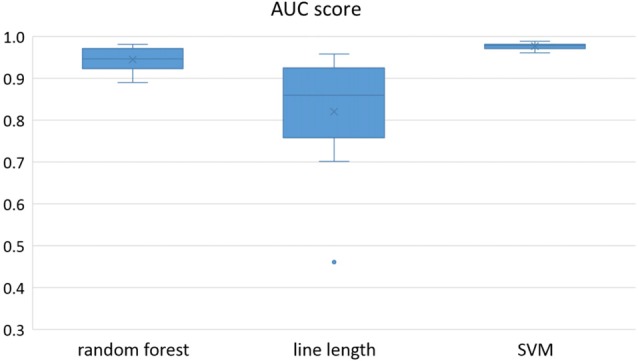
Detection performance of the classifiers over all the patients in multichannel mode.

**Figure 4:**
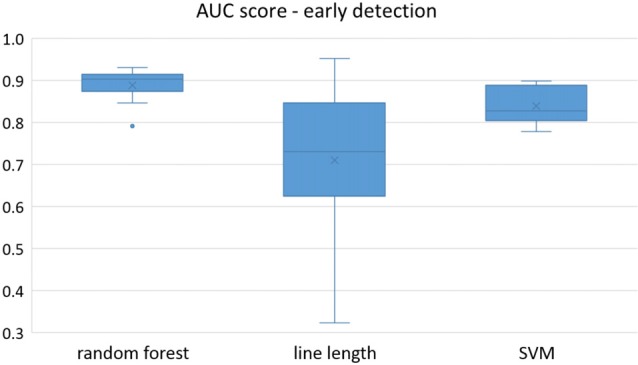
Early detection performance of the classifiers over all the patients in multichannel mode. Here, just the seizures which could be detected in the first 10 s are counted as a true positive.

Owing to the inevitable trade-off between detection performance of classifiers in terms of the sensitivity and specificity, a higher sensitivity can be achieved if lower specificity is accepted. As a result, to have a threshold free comparison of the classifiers, receiver operating characteristic (ROC) curves were generated with the area under the ROC curve (AUC) as a performance parameter. ROC curve is a graphical plot that illustrates how the diagnostic ability of a binary classifier as its discrimination threshold is varied. It is created by plotting the fraction of True Positives (Y-axis) vs. the fraction of False Positives (X-axis). For a binary classifier, the AUC is equivalent to the probability that the classifier will rank a randomly chosen positive instance higher than a randomly chosen negative instance. For every patient, we selected one channel with the highest AUC. This was done separately for each classifier. Next, we compared the average AUC of the selected channels over all the patients between classifiers (Figure 1). Since the detection delay is not projected in AUC, we measured a second AUC for seizures that were detected within the first 10 s of seizure onset as marked in the EEG, and named it early seizure detection (Figure 2). The average AUC of classifiers over all the patients in multichannel mode is compared in Figure 3. The same classifier comparison for early seizure detection in multichannel mode was done in Figure 4. Subsequently, we compared the mean and median values of AUC between the classifiers over all the patients (Table 1). As the single channel classifier, Random Forest classifier with mean AUC score of 0.9 (median 0.89) had the best performance. The second best was the SVM classifier with a mean AUC score 0.88 (median 0.88) in comparison to Line length classifier with a mean AUC score of 0.83 (median 0.86). The same comparison was also performed for early seizure detection (Table 1). Again, the Random Forest classifier with a mean AUC score of 0.83 (median 85%) had the best performance followed by Line length classifier with mean AUC score of 0.73 (median 75%) and SVM with a mean AUC score of 0.71 (median 0.72). This shows that the Random Forest classifier is able to provide early seizure detections with a high sensitivity. In the case of SVM, the decrease of the AUC score for early seizure detection indicates that it has relatively longer detection delay in comparison to the other two classifiers. In the case of multichannel classification, the results are different (Table 2). SVM with mean AUC score of 0.98 (median 0.98) had the best performance. The second-best classifier is the Random Forest classifier with 0.95 (median 0.95) in comparison to Line length classifier with mean AUC score of 0.82 (median 0.86). The results of early seizure detection are to some extent different from when the whole seizure is used for testing (Table 2). In these cases the Random Forest classifier had the best performance with mean AUC score of 0.89 (median 0.90), followed by the SVM classifier with a mean AUC score of 0.84 (median 0.83) and finally the Line length classifier with mean AUC score of 0.71 (median 0.73).

**Table 1:** Comparison of the area under the receiver operating characteristic (ROC) curve (AUC) over all the patients between the classifiers in a single-channel mode.

AUC			
	Random Forest	SVM	Line length
Mean	0.90	0.88	0.83
Median	0.89	0.88	0.86
**AUC for early detection**			
	**Random Forest**	**SVM**	**Line length**
Mean	0.83	0.71	0.73
Median	0.85	0.72	0.75

**Table 2:** Comparison of the AUC over all the patients between the classifiers in multichannel mode.

AUC			
	Random Forest	SVM	Line length
Mean	0.95	0.98	0.82
Median	0.95	0.98	0.86
**AUC for early detection**			
	**Random Forest**	**SVM**	**Line length**
Mean	0.89	0.84	0.71
Median	0.90	0.83	0.73

**Figure 5:**
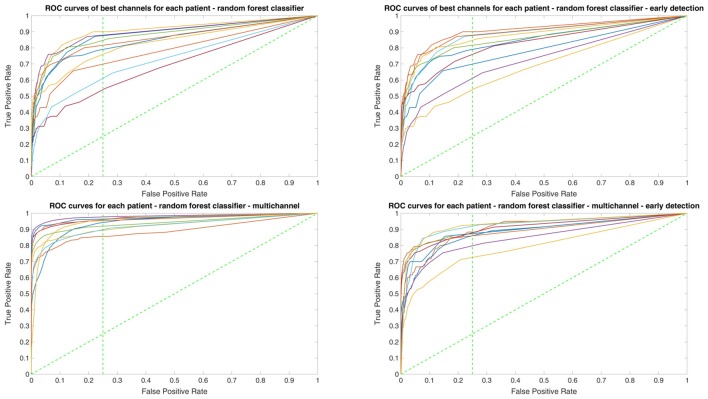
ROC curves of all patients in single and multichannel mode for the Random Forest classifier. The dashed line separates the early retrieval area.

**Figure 6:**
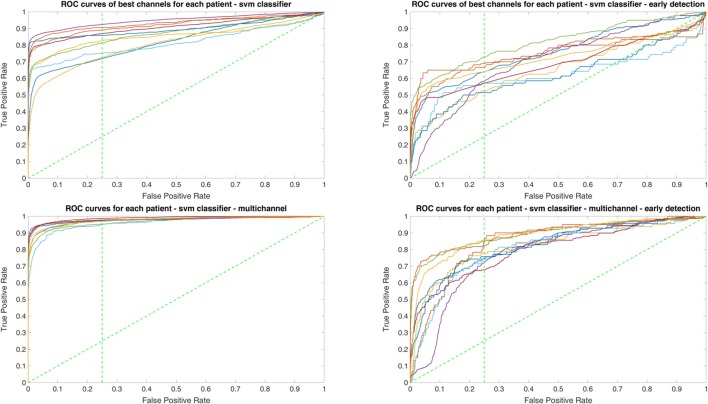
ROC curves of all patients in single and multichannel mode for the support vector machine (SVM) classifier. The dashed line separates the early retrieval area.

**Figure 7:**
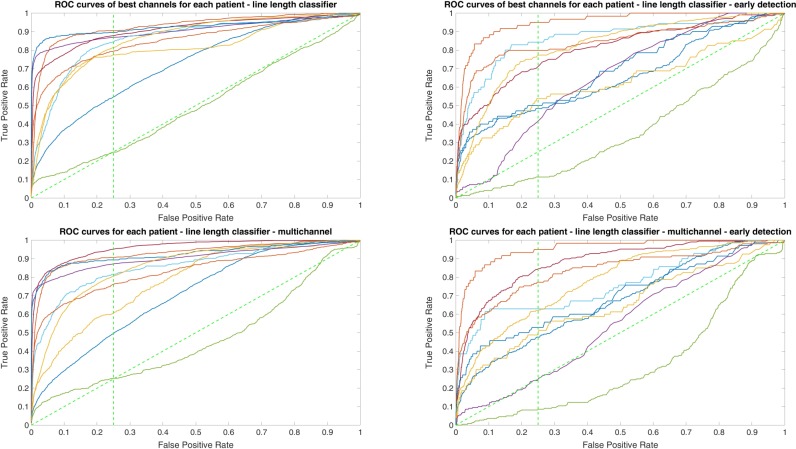
ROC curves of all patients in single and multichannel mode for the line length classifier. The dashed line separates the early retrieval area.

Since two ROC curves with the same AUC value can be quite different, it is also important to check the actual curves, especially when evaluating the results. Therefore, we plotted the ROC curves of the three classifiers. For each classifier there are four plots: (1) AUC for the seizure detection in single channel mode; (2) AUC for early seizure detection in single channel mode; (3) AUC for the seizure detection in multichannel mode; and (4) AUC for early seizure detection in the multichannel mode. The Random Forest classifier ROC curves are plotted in Figure 5, SVM classifier in Figure 6 and line length classifier in Figure 7.

### Hardware Implementation of Superior Machine Learning Algorithm

**Figure 8:**
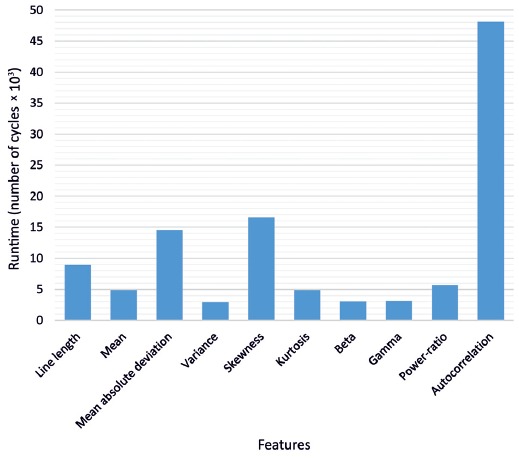
Required runtime in number of cycles for each feature.

Regarding feature calculation, the runtime in clock cycles for each feature is shown in Figure 8. Due to the more complex calculation required for the autocorrelation, almost half of the cycles were used for this feature. In total, 112,773 cycles were required to perform the feature extraction for one channel, yielding in a power consumption of 30 μW per channel.

Regarding classifier implementation as mentioned in the previous section, the mean AUC for early seizure detection of all the patients for the Random Forest was 83% (median 85%) and 71% (median 72%) for the SVM in the single channel mode. In the multichannel mode, the Random Forest classifier with a mean AUC of 0.89 (median 0.90) outperformed SVM again with mean AUC of 0.84 (median 0.83). Therefore, due to the superior performance and lower complexity of the Random Forest classifier in comparison to SVM, it was selected for the low power implementation on the microcontroller to evaluate its energy efficiency as the seizure detection module for implantable devices.

The size of the classifier with respect to the number of branches of the trees depends on the size of the training set. Since the required memory is beyond the microcontroller’s internal memory of 256 kB, an external 8 MB low power flash memory was used. To represent a node of a tree only 5 bytes were required: 2 bytes for the threshold, 2 bytes to address the child node in a leaf for the label, and 1 byte for the feature. After calculating votes for each tree, the number of non-seizure votes was counted. To save runtime and energy, after 50 negative votes out of 100 votes, vote counting was stopped and the next electrode was processed. The exact power consumption depends on the number of trees, which have to be evaluated until a decision about the occurrence of a seizure can be made. For the 10 patients in the used dataset, between 1,025 nodes and 1,441 nodes per electrode were evaluated every second for classification, resulting in a total power consumption of 174 μW to 245 μW per channel.

## Discussion and Outlook

There are several research groups focusing on optimizing seizure detection algorithms to be applied in a responsive neurostimulation implant device for the treatment of epilepsy. Similar to our approach, (Truong et al., 2017) used the random forest classifier for seizure detection. They focused on the number of channels required for seizure detection and introduced an automatic channel selection method. Automatic channel selection is an interesting option to adapt a responsive system to long-term alterations in the patterns of seizure generation. This may allow for adaptive changes related to the localization and even lateralization (see for example Spencer et al., 2011; Smart et al., 2013 and King-Stephens et al., 2015) of the SOZ over longer periods of time.

In our study, the number of channels considered for seizure detection was limited to 1–4 expert selected channels with the aim to develop a classifier that can be implemented on ultra-low power hardware. The channels were selected based on the earliest visibility of an ictal epileptic pattern. We compared the classifiers in two different settings (single and multichannel mode) to see how increasing the number of detection channels affects the classifier performance. The lower number of channels as compared to the study of Truong et al. (2017) results in a much lower computationally demanding classifier. Depending on patient characteristics, however, the additional implementation of automatic channel selection may be a valuable future extension of our proposed system. In another study, Osorio et al. (2002) suggested a generic algorithm for application in a closed loop system. Recent benchmarks for seizure detection based on intracranial EEG are given in a report of a crowdsourcing competition by Baldassano et al. (2017). As in our study, the average of the AUCs for seizure and early seizure classification were used as performance metrics. In comparison, our study had more methodological restrictions. First, we used only four channels; whereas in crowdsourcing competition no limitation on the number of channels was set; therefore, a higher number of channels for seizure detection (around 35 channels for the winner classifier) were used. Besides, in the definition of early seizure detection; we selected a time limit based on a window of the first 10 s from visual seizure onset, while in crowdsourcing competition this limit had been chosen as 15 s. The best performing algorithm of the competition (also using random forest classifier with a high dimension of features) had an AUC around 0.96; while our random forest classifier had an AUC 0.92, which may be related to the more restricted boundary conditions of our study. So far, major progress in the field was achieved with the FDA approved responsive neurostimulation implant device, which is available for clinical applications. The RNS system from Neuropace Inc. (Sun and Morrell, 2014), which is the first intelligent implant for continuously analyzing ongoing brain activity. The RNS system uses line length, area, and bandpass as features. The bandpass detector is similar to the one described by Gotman (1982). For each of two sensing channels, up to two independent detectors can be applied (Sun and Morrell, 2014). After using the RNS device for 2 years in patients Heck et al. (2014) reported, that the RNS system stimulates the brain about 5.9 m/day, and each stimulation has a burst duration of 100 ms, in contrast to an average reported seizure frequency of 33.5/month. This indicates approximately more than 3,000 false detections per correct stimulation, similar to the low specificity found in our analyses on long-term data from the European epilepsy database. Unfortunately, due to the lack of quantitative information provided by the Neuropace, a precise assessment of the RNS system is not possible (Osorio, 2014).

Both, a wider spectrum of extracted features and an advanced classification of ictal electrographic patterns came closer to a true closed-loop intervention strategy. After implementing such a system, a comparison of the overall performance of the three classifiers showed that the Random Forest classifier, due to its higher AUC for early seizure detection in both single and multichannel mode is a better option for closed-loop application in comparison to SVM. However, in offline applications, where the detection delay is not important, multichannel SVM outperforms the other classifiers. Besides, comparison of single channel and multichannel classification results of Random Forest classifier and SVM classifier, shows that the number of detection channels has a profound influence on the quality of the classification results encouraging the evaluation of multichannel approaches deeper in future studies.

We furthermore showed that Random Forest classification is feasible on a low power microcontroller. We decided to implement the detector on a microcontroller due to its easier application as well as keeping the power consumption low. For this implementation, computation of the features needs simplification and an efficient programming of the microcontroller using its internal DMA-controller, hardware multiplier and a low energy DSP accelerator instead of the more power-demanding CPU. Since this implementation is on a microcontroller, it is difficult to compare it to the computational efficiency of other approaches as the one performed in the study of Truong et al. (2017). They showed a superior efficiency to another state-of-the-art approach, but it can be inferred that due to the lower number of channels, smaller number of trees in our classifier and lower feature dimensionality, the computational load of our design is lower. Presenting the number of cycles needed for feature calculation and the range of the needed power for the random forest classifier are closer to the design approaches for implantable devices.

Research in the area of closed loop devices using intracranial EEG has many applications ranging from treating diseases like Parkinson, tremor and epilepsy, to restoring efficient communication, and movement ability to those suffering from paralysis and rehabilitation. This technology is advancing beyond preclinical studies, with trials beginning in human patients. Moving from open loop approaches (Mehring et al., 2004; Milekovic et al., 2012; Pistohl et al., 2012) to responsive systems is an important step to tailor and fine-tune the application of this technology (Rosin et al., 2011; Priori et al., 2013; Tabot et al., 2013). Concepts and implementations in this study are partly specific for their application in seizure detection, especially the selection of features driven by typical EEG patterns at seizure onset. Nevertheless, it can be shown that the combination of features in the time and frequency domains and a classifier that is successfully applied on offline data using a general-purpose computer can be transferred to a low power implementation using a microcontroller suitable for an implant. The translation to other treatment areas would primarily require carefully selecting features representing properties of target signals and estimating their computational costs. Thus, a foundation is set to open new frontiers in treatment and rehabilitation based on implantable devices.

## Ethics Statement

The study was approved by the Ethics Committee at the Freiburg University Medical Center.

## Author Contributions

All authors made major contributions to the manuscript. FM programmed feature extraction and classification algorithms, performed data analyses using standard workstations and contributed to the writing of the manuscript. SH performed experiments and calculations with microcontrollers as well as microcontroller-based data analyses, and contributed to the writing of the manuscript. MD gave advice in the design of seizure detection, contributed to the EEG analyses and to the writing of the manuscript. PW designed the hardware implementation and contributed to the writing of the manuscript. AS-B designed the interdisciplinary project, contributed to EEG selection for analyses and to the writing of the manuscript.

## Conflict of Interest Statement

The authors declare that the research was conducted in the absence of any commercial or financial relationships that could be construed as a potential conflict of interest. The handling Editor declared a shared affiliation, though no other collaboration, with the authors (FM, SH, MD, PW and AS-B), and the handling Editor states that the process met the standards of a fair and objective review.
